# Malignant hypercalcemia as the debut of acute lymphoblastic leukemia in a pediatric patient—a diagnostic and therapeutic approach: Case report

**DOI:** 10.3389/fped.2022.1027421

**Published:** 2022-11-28

**Authors:** Carolina Bonilla Gonzalez, Sarha M. Vargas Muñoz, María Luisa Contreras Diaz, Evelyn Obando Belalcazar, Camila Uribe

**Affiliations:** ^1^ Pediatric Intensive Care Unit, Department of Pediatrics, Fundación Santa Fe de Bogotá University Hospital, Bogotá, Colombia; ^2^ Department of Pediatrics, Fundación Santa Fe de Bogotá, Bogotá, Colombia; ^3^ Department of Pediatrics, Fundación Santa Fe de Bogotá University Hospital, Universidad de los Andes, Bogotá, Colombia

**Keywords:** child, hypercalcemia, hematologic neoplasms, leukemia, zoledronic acid

## Abstract

**Background:**

Hypercalcemia is a rare metabolic disorder in the pediatric population, with several differential diagnoses that resemble hematologic malignancies. In cases of severe hypercalcemia, therapeutic strategies other than hyperhydration, such as the use of bisphosphonates, have been described.

**Case presentation:**

We present the case of a previously healthy 12-year-old boy who was admitted to the emergency department due to fatigue, hypo-responsiveness, and progressively worsening poor appetite for the previous 19 days. Initial laboratory tests revealed severe hypercalcemia (total calcium: 19 mg/dl), hyperphosphatemia, elevated creatinine, and hyperuricemia. Management with hyperhydration and xanthine oxidase inhibitor (allopurinol) was provided. The patient was transferred to the pediatric intensive care unit where treatment with furosemide, systemic corticosteroid, and zoledronic acid was started. Metabolic, infectious, renal, and endocrinological causes were excluded. Follow-up paraclinical studies showed a progressive hematologic involvement with heterogeneous hypochromic microcytic anemia, thrombocytopenia, and elevated lactic dehydrogenase. Bone marrow aspiration and biopsy were performed, which confirmed the diagnosis of B-precursor acute lymphoblastic leukemia. Hypercalcemia was resolved 72 h after the application of bisphosphonates.

**Conclusion:**

Hypercalcemia as an oncological metabolic emergency in the onset of acute lymphoblastic leukemia is uncommon in children. The use of intravenous bisphosphonates is an effective therapy in the early resolution of the condition. We present the case of a 12-year-old patient with malignant hypercalcemia who responded favorably to the use of a single dose of bisphosphonates.

## Background

Hypercalcemia is a rare metabolic disorder in the pediatric population. There are different associated etiologies (metabolic, nutritional, drug-induced, genetic, endocrinological, or neoplastic) (1). Malignant hypercalcemia can occur in 0.4%–0.7% of cases as a complication of neoplastic pathology, among which acute lymphoblastic leukemia is frequently found in the initial stage of the disease (2). This finding represents an oncological emergency with an associated risk of mortality; therefore, a timely diagnosis and approach are necessary to minimize organic damage (3). The initial treatment consists of the control of the underlying pathology. However, in cases of severe hypercalcemia, therapeutic strategies such as hyperhydration and the use of bisphosphonates have been described in adults (1). We present the case of a 12-year-old patient who showed malignant hypercalcemia associated with hyperuricemia and acute renal failure as part of the onset of acute lymphoblastic leukemia and who was provided pharmacological treatment with zoledronic acid, an understudied management in the pediatric population.

## Case presentation

A 12-year-old male patient, with a non-relevant medical history, consulted for 19 days of progressive hypo-responsiveness and hyporexia, with clinical decline and emesis, in the last days before admission. The mother reported a loss of 5 kg in the patient in the last 15 days. At the initial consultation, they reported severe hypercalcemia of 19 mg/dl (>14.5 mg/dl), hyperphosphatemia (6.6 mg/dl), elevated creatinine (1.45 mg/dl) as well as hyperuricemia (9.3 mg/dl) (Table 1). An x-ray of the long bones showed a lesion with a periosteal reaction of lytic characteristics in the medial distal metaphysis of the right femur. Under the context of tumor lysis syndrome, hyperhydration and management with allopurinol were started.

**Table 1:** Laboratory tests over time.

Blood test	Reference values	Day 1 out-of-hospital	Day 1 admission/PICU	Day 2	Day 3	Day 4
Serum test						
Sodium (mmol/L)	136–145	144	141.55		142.2	139.35
Potassium (mmol/L)	3.4–4.7	3.89	↓2.89	↓2.9	↓3.01	↓2.5
Phosphorus (mmol/L)	1.3–1.9	↓1.12	1.45	↓1.06	1.41	↓0.9
Magnesium (mmol/L)	0.86–1.17	0.97	↓0.7	↓0.69	↓0.7	↓0.64
Chlorine (mmol/L)	100–107	101	104.23		100.8	100
Ionic calcium (mmol/L)	1.22–1.37		↑2.35	↑1.87	↑1.69	1.02
Calcium (total) (mmol/L)	2.3–2.6	↑4.71	↑3.99	↑3.99	↑2.94	2.2
Uric acid (mmol/L)	0.2–0.5	↑0.55	0.46		<0.08	<0.08
Creatinine (mcmol/L)	39.78–71.61	↑128.18	↑121.11		↑130.83	↑83.09
Urea nitrogen (mmol/L)	2.6–7.5	↑9.03	↑9.2		↑15.35	↑11.32
Leukocytes ×10^3^/μl	4.5–13.5	8.160	5.600		↓4.900	↓4.100
Neutrophils (%)	33–76	55	57.8		86	78.3
Lymphocytes (%)	15–55	36	38		17	20.4
Monocytes (%)	0–5	4	3.3		0	1.2
Eosinophils (%)	0–3	0	0		0	0
Hemoglobin (g/dl)	12–16	14.6	↓10.9		↓10.9	↓9.1
Hematocrit (%)	36–45	42.8	↓33.9		↓33.2	↓29
Mean corpuscular volume (fl)	76–90	↓72.8	77.8		77.6	77.8
Corpuscular mean hemoglobin (fmol/cell)	0.39–0.54	1.54	1.56		1.58	1.52
Platelets (10^3^/ml)	150–350	326	207		↓95	188
Blasts (%)	0				↑7	
25-Hydroxy vitamin D (nmol/L)	≥50	73.63				
Parathyroid hormone (pmol/L)	1.6–6.9	↓0.65	↓0.48			
Thyroid stimulating hormone (mIU/L)	0.45–4.5	1.7	1,485			
Free thyroxine (FT4) (pmol/L)	14.2–25.7	17.63	14.61			
Total bilirubin (mcmol/L)	1.71–11.97		4.1			
Bilirubin (conjugated) (mcmol/L)	0.86–4.96		4.1			
Prothrombin time (PT)	12.7–16.1		11.3/11.1		11.4/10.9	
International normalized ratio (INR)	0.97–1.3		1.04		1.13	
Alkaline phosphatase (U/L)	141–260		211			
Lactic dehydrogenase (U/L)	170–283	↑685	↑522		↑453	↑406
Lactate (arterial) (mmol/L)	1.04–2.4	2.1	1.2	↑3.3		
C-reactive protein (mg/dl)	0.1–1.7		↑9.579			
Urine test						
Ca/creatinine ratio (mg/mg)	<0.21	↑0.7	↑0.93			

Upon admission to our institution, the patient presented normal vital signs and appropriate anthropometry for his age. Mucocutaneous paleness was the only abnormal finding. Hyperhydration was continued and management with a single dose of rasburicase was indicated. A hypercalcemic crisis with kidney involvement secondary to volume depletion was confirmed, with acute kidney injury RIFLE I and glomerular filtration rate (GFR) at 48.8 ml/min/1.73 m^2^. Management with furosemide was added, and given the severe levels of hypercalcemia, methylprednisolone and bisphosphonate (a single dose of zoledronic acid 0.05 mg/kg/IV) were added.

Studies were performed for possible infectious, metabolic, renal, endocrinological, and neoplastic etiologies associated with acquired hypercalcemia (Table 1). A malignant bone neoplasm was initially ruled out; magnetic resonance imaging of the lower limbs only showed a lesion compatible with osteochondroma, discarding a possible malignant bone neoplasm. Paraclinical follow-up tests showed heterogeneous hypochromic microcytic anemia, with a slight decrease in platelets, without compromise of other cell lines and elevated lactic dehydrogenase (Table 1).

Endocrinological studies were expanded; parathyroid hormone (PTH) levels were found to be in the suboptimal range, calcitriol was normal, and 25-hydroxyvitamin D3 levels were optimal. As the patient had no history of drug use to justify serum calcium levels and had not presented any state of prolonged immobilization, severe PTH-independent hypercalcemia was diagnosed. Among secondary causes, thyroid function disorders were ruled out by normal thyroid ultrasound. Normal alkaline phosphatase levels and reported imaging studies made the bone origin of hypercalcemia less likely.

Regarding the nephrological study, the abdominal ultrasound was not suggestive of nephrocalcinosis, as evidenced by the hypercalciuria (a slightly elevated calcium/creatinine ratio of 0.7), which was justified by the severity of the hypercalcemia, making a tubular reabsorption disorder unlikely.

There were no clinical or paraclinical signs of infection; the elevated level of C-reactive protein (9 mg/dl) was justified by acute inflammation of unknown etiology. The patient persisted with anemia and elevation of indirect bilirubin, ruling out hemolytic etiologies. A subsequent paraclinical follow-up showed thrombocytopenia in addition to heterogeneous hypochromic microcytic anemia. The control blood count reported 7% immature cells, which had not previously been discovered. Lactate dehydrogenase (LDH) levels remained high (453 U/L). Suspicion of the lymphoproliferative syndrome was considered; a biopsy and a bone marrow aspirate were indicated on the third day of hospital admission.

The biopsy report demonstrated precursor B acute lymphoblastic leukemia, of intermediate-risk, central nervous system (CNS) 1 status. Translocations (4; 11), BCR/ABL (12; 21), and 46 XY leukemic karyotype were all ruled out, and therapy according to the Acute Lymphoblastic Leukaemia Intercontinental 2009 protocol was subsequently started. The patient continued multidisciplinary management and his renal function normalized on the fourth day of admission, reaching a GFR of 71.17 ml/min/1.73 m^2^. He never developed any additional complications beyond acute renal failure secondary to the hypercalcemic crisis. He completed treatment with allopurinol for 7 days, achieving normal uric acid levels from the second day of admission and his calcium levels normalized 72 h after the application of zoledronic acid, subsequently requiring replacement due to hypocalcemia without additional severe adverse reactions (Table 2).

**Table 2:** Calcium (total mmol/L) behavior after zoledronic acid administration.

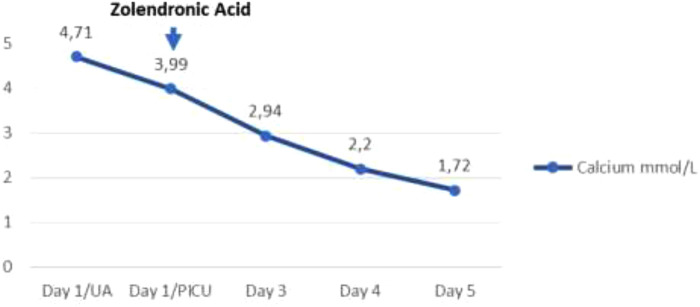

## Discussion

We present an unusual debut of a hematologic malignancy and the efficacy of a non-common therapy in the pediatric population, such as the use of bisphosphonates. Hypercalcemia is a rare metabolic disorder in children. Its etiology in the pediatric population is different from adults and includes immobilization, granulomatous disorders (e.g., tuberculosis, sarcoidosis, and cat-scratch disease), hyperparathyroidism, hypervitaminosis D and/or A, endocrine disorders (e.g., thyrotoxicosis, pheochromocytoma, and adrenal insufficiency), and medications (e.g., thiazide diuretics and hypocalciuric) among others (1, 4). Hypercalcemia can be present in approximately 0.5%–1.5% of malignancies (5). In adults, it is mostly described in solid tumors (6) and in some rare situations associated with acute leukemia or chronic myeloid leukemia, mainly in the accelerated phases of the disease or blast phase (6, 7).

The association between hypercalcemia with leukemia was first recorded in 1956 by Myers (4). Further case reports and studies in children were later recorded, including a study conducted in the United Kingdom between 2003 and 2014 that showed that although hypercalcemia can occur in any type of cancer, it was more common in children with leukemia (8). Other studies have shown the association of malignant hypercalcemia with precursor phenotype B leukemia, associated with a leukocyte count of less than 20,000/mm^3^, and hypercalcemia at the time of diagnosis, as it presented itself in our case (6). Currently, it is suggested that the presence of hypercalcemia in children with leukemia does not affect their prognosis (9).

Severe hypercalcemia can be associated with a wide spectrum of clinical manifestations depending on the severity and chronicity of the condition (4). Its clinical presentation can generally have an insidious onset of a few weeks. The most common findings are lethargy, hypotonia, anorexia, weight loss or growth retardation, polydipsia, polyuria, vomiting, bone pain, constipation, and abdominal pain (3, 10). Severe cases may develop kidney failure, pancreatitis, and compromised consciousness. The mechanism leading to the renal concentration defect involves tubulointerstitial injury by calcium deposits in the marrow, the downregulation of the water channel of aquaporin-2 (calcium-induced renal resistance to arginine vasopressin), and the activation of calcium-sensitive receptors (10). In addition, as in the case of our patient, a significant increase in uric acid may occur, with the precipitation of its crystals at the renal tubular level associated with direct injury to the tubular epithelial cells and indirect injury through renal vasoconstriction able to generate interstitial tubule injury with the consequent recruitment of pro-inflammatory cytokines and tissue damage (2).

The finding of severe hypercalcemia (>14.5 mg/dl) is defined as a hypercalcemic crisis, which is typically symptomatic and represents a medical emergency. CNS dysfunction or cardiovascular alterations are, together with kidney failure, the main causes of mortality in these patients, forcing immediate corrective measures to be taken for the restoration of homeostasis and the correct functioning of the compromised organs (11).

**Figure 1:**
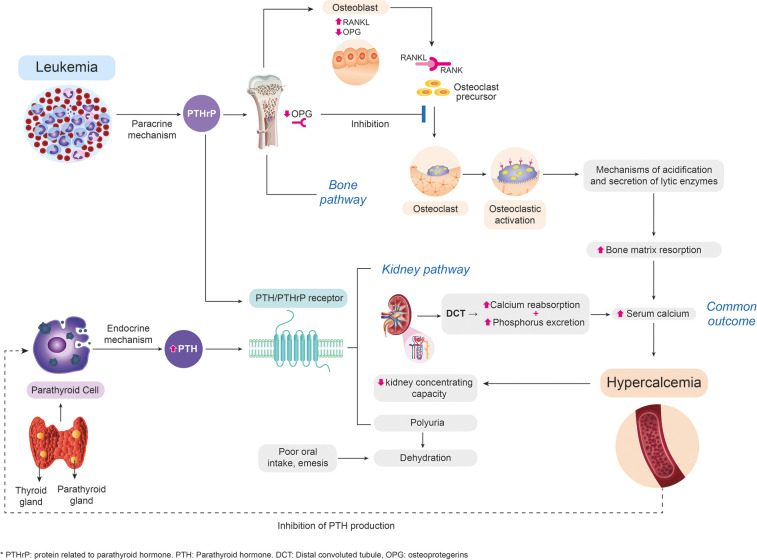
Approach to the pathophysiological way to hypercalcemia of malignancy. Design and flowchart intellectual property by the authors. This image was developed based on the actual related scientific literature. PTHrP, secreted systemically by malignant para-endocrine pathways, shares a close homology with PTH. At the renal level, PTHrP stimulates calcium reabsorption in the distal convoluted tubule (1, 4). Elevated calcium levels have an inhibitory action on the production of PTH by the parathyroid, which explains the abnormally decreased serum levels of PTH. Additionally, patients with hypercalcemia present a compromise of the renal concentration capacity that causes polyuria with volume depletion, which, together with the decrease in oral intake, associated nausea, and emesis, leads to the development of acute renal failure (14). At the bone level, PTHrP stimulates osteoblasts to secrete regulatory hormones such as the ligand of the activating receptor for nuclear factor-kB (RANKL) (12), which binds to the osteoclast membrane receptor favoring signaling pathways, their differentiation, and the resorption of the bone matrix through the generation of mechanisms of acidification and secretion of lytic enzymes, and inhibits the production of osteoprotegerin (inhibitor of differentiation of osteoclasts), perpetuating the process of bone resorption (15). PTH, parathyroid hormone; PTHrP, parathyroid hormone-related protein.

During the first clinical approach, it is important to determine whether it is a PTH-dependent or PTH-independent cause. When hypercalcemia is independent of PTH, primary (genetic) and secondary causes such as malignancy should be studied (4, 10). The mechanism of hypercalcemia secondary to malignancy varies according to the type of oncological pathology. Different related pathophysiological mechanisms have been described; however, we highlight the mechanism of humoral malignant hypercalcemia, which could be related to the case presented as it explains the findings of suppressed PTH, low levels of 1,25-dihydroxy vitamin D, and slightly increased serum phosphorus. The mentioned mechanism is responsible for 80% of the causes of hypercalcemia (12), and it is caused by ectopic systemic secretion of parathyroid hormone-related protein (PTHrP) from malignant tumors and has been reported in acute lymphoblastic leukemia. PTHrP has actions similar to PTH and results in increased bone resorption and renal calcium reabsorption (9, 10, 13) (Figure 1).

Treatment of hypercalcemia depends on the underlying disease (10). The urgency and aggressiveness of management are related to the severity of hypercalcemia (9). In cases of severe hypercalcemia, as seen in our patient, immediate treatment with appropriate hydration is required, trying to achieve two goals, the restoration of renal flow and the decrease in urinary density, minimizing the precipitation of crystals (16).

The excretion of urinary calcium can be increased by inhibiting sodium reabsorption from the proximal tubule and the Henle loop, thus reducing passive calcium reabsorption (9). Proximal resorption is inhibited by volume expansion through an intravenous (IV) saline infusion, which increases sodium, calcium, and water delivery to the loop of Henle and the administration of a loop diuretic such as furosemide that blocks transport at this level (9). Most children with severe hypercalcemia have volume contraction secondary to decreased fluid intake and the natriuretic effect of hypercalcemia; therefore, loop diuretics should be used with caution as they will increase the chances of developing nephrocalcinosis (9, 10, 16).

Glucocorticoids inhibit the conversion of 25OHD to its active metabolite, 1,25 (OH) 2D, decrease intestinal calcium absorption, and may be useful in hematologic malignancy (10). Calcitonin inhibits osteoclastic bone resorption and, to a lesser extent, increases renal calcium excretion. It has a rapid onset of action; however, it has a short-lasting effect, and can cause tachyphylaxis after 48 h (16).

IV bisphosphonates, such as pamidronate and zoledronic acid, are approved only for adults by the US Food and Drug Administration (16). They provide a slow onset of action and a more sustained effect (10), acting more slowly than calcitonin and intravenous fluids, achieving initial calcium decreases in 2–4 days and nadir levels in 4–7 days (16). These are adsorbed on the surface of bone hydroxyapatite and act by blocking osteoclast-mediated bone resorption, rapidly reducing serum calcium levels. The effects can last for weeks, and they are the most effective agent for children with malignant hypercalcemia (9, 10).

Zoledronic acid is considered more potent, effective, and convenient than pamidronate. Zoledronic acid in a single dose of 4 mg as an IV infusion over at least 15 min is recommended for the initial treatment of malignant hypercalcemia (9). However, some case reports have shown effective doses from a single dose of 0.025 mg/kg (17), 2 mg/kg (6), or 4 mg in the infusion of 30 min with 100 ml of isotonic saline solution (9). The adverse effects of zoledronic acid are common in the days after its administration and include acute phase reactions (flu-like symptoms), hypocalcemia, hypophosphatemia, and, less frequently, kidney damage; hence, monitoring and management of these complications must be strict and periodic (9).

Although the number of reports of its use in patients with leukemia is limited (4), the present case shows that a successful resolution of hypercalcemia is presented within 72 h of the use of zoledronic acid at a single IV dose of 0.05 mg/kg, with the only adverse reaction presented being hypokalemia found after a follow-up of paraclinical tests, handled with substitution and resolved without complications. In cases of hypercalcemia resistance to the treatments described or seriously compromised kidney function, which may be life-threatening, hemodialysis against a low calcium dialyzer is more effective than peritoneal dialysis (10, 18).

## Conclusion

Hypercalcemia is an oncological metabolic emergency in the onset of acute lymphoblastic leukemia and an uncommon presenting sign in children. In addition to management with rehydration, it has been established that the use of IV bisphosphonates is an effective therapy in the early resolution of the condition. Despite the poor evidence on the dosage of zoledronic acid in children, we consider that it could represent an effective alternative in management. We present the case of a 12-year-old patient with malignant hypercalcemia who responded favorably and quickly to the use of zoledronic acid at a dose of 0.5 mg/kg in a single dose.

## Data Availability

The original contributions presented in the study are included in the article/Supplementary Material, further inquiries can be directed to the corresponding author/s.
